# Whole-genome DNA methylome analysis of different developmental stages of the entomopathogenic fungus *Beauveria bassiana* NCHU-157 by nanopore sequencing

**DOI:** 10.3389/fgene.2023.1085631

**Published:** 2023-01-18

**Authors:** Yi-Hsuan Li, Ju-Chun Chang, Ming-Ren Yen, Yu-Feng Huang, Tzu-Han Chen, Li-Hung Chen, Yu-Shin Nai

**Affiliations:** ^1^ Department of Entomology, National Chung Hsing University, Taichung City, Taiwan; ^2^ Department of Computer Science and Engineering, Yuan-Ze University, Taoyuan City, Taiwan; ^3^ Department of Plant Pathology, National Chung Hsing University, Taichung City, Taiwan; ^4^ Advanced Plant Biotechnology Center, National Chung Hsing University, Taichung City, Taiwan

**Keywords:** Oxford Nanopore Technologies (ONT), entomopathogenic fungi (EPF), Beauveria bassiana, DNA methylation, differential DNA methylated regions (DMR)

## Abstract

The entomopathogenic fungus (EPF), *Beauveria bassiana,* is an important and commonly used EPF for microbial control. However, the role of DNA methylation has not been thoroughly studied. Therefore, the whole genomic DNA methylome of one promising EPF isolate, *B. bassiana* NCHU-157 (Bb-NCHU-157), was investigated by Oxford Nanopore Technologies (ONT). First, the whole genome of Bb-NCHU-157 was sequenced by next-generation sequencing (NGS) and ONT. The genome of Bb-NCHU-157 contains 16 contigs with 34.19 Mb and 50% GC content, which are composed of 10,848 putative protein-coding genes. Two putative DNA methyltransferases (DNMTs) were found, including Dim-2 and C-5 cytosine-specific DNA methylases. Both DNMTs showed higher expression levels in the mycelium stage than in the conidia stage, indicating that development of DNA methylation in Bb-NCHU-157 might occur in the mycelium stage. The global methylation level of the mycelium stage (5 mC = 4.56%, CG = 3.33%, CHG = 0.74%, CHH = 0.49%) was higher than that of the conidial stage (5 mC = 2.99%, CG = 1.99%, CHG = 0.63%, CHH = 0.37%) in both the gene and transposable element (TE) regions. Furthermore, the TE regions showed higher methylation frequencies than the gene regions, especially for CHH site methylation, suggesting regulation of genomic stabilization during mycelium development. In the gene regions, high methylation frequencies were found around the transcription start site (TSS) and transcription end site (TES). Moreover, CG and CHG methylation mainly occur in the promoter and intergenic regions, while CHH methylation occurs in the TE region. Among the methylated regions, 371, 661, and 756 differentially DNA methylated regions (DMRs) were hypermethylated in the mycelium in CG, CHG, and CHH, while only 13 and 7 DMRs were hypomethylated in the mycelium in CHG, and CHH, respectively. Genes located in the DMR shared the GO terms, DNA binding (GO: 0003677), and sequence-specific DNA binding (GO: 0043565) for hypermethylation in the mycelium, suggesting that methylation might regulate gene expression from the initial process. Evaluation of the DNA methylome in Bb-NCHU-157 by ONT provided new insight into this field. These data will be further validated, and epigenetic regulation during the development of *B. bassiana* will be explored.

## Introduction

Entomopathogenic fungi (EPFs) have shown insect pathogenicity and dwell in the soil when they live without their hosts. They are the natural enemies of insect pests and can kill or seriously disable them (Sharma et al., 2018). Some show distinct ecomorphological adaptations to the life cycles of their hosts and produce resting spores to survive adverse conditions. Most of them belong to the Ascomycota and Zygomycota phyla. For both of these groups, contact can cause arthropod infection. In Ascomycota, the genera *Beauveria*, *Metarhizium*, *Isaria*, *Lecanicilium*, *Nomuraea*, *Hirsutella* and *Paecilomyces* have been studied extensively (Kim et al., 2018; Chang et al., 2021; Liu et al., 2021). They can form natural epizootics in some pest populations.

EPFs can be used to develop microbial control agents. They are toxic or pathogenic only to a single class or single pest and are harmless to beneficial species, humans and non-standard organisms. They can also avoid ecological balance and provide another safe, economical and effective option. For example, *Beauveria bassiana* has been used as an environmentally friendly alternative chemical insecticide (Gao et al., 2011; Furuie et al., 2022). As mentioned above, the importance of fungi as microbial control agents has created great interest in the regulation of the formation of these products. Knowledge about how epigenetic mechanisms influence regulatory processes consequently initiated investigations about the interplay of epigenetics and product formation by using entomopathogenic fungi.

DNA methylation plays an important role in many biological processes. Previous research showed that it was related to the promoter regions and transcribed regions of genes, transposable elements, and repeat sequences in both animals and plants (Li et al., 2017). In contrast, it is difficult to detect DNA methylation levels in most fungi (Wang et al., 2017). In different fungal species, their genome methylation patterns may also not be strictly conserved. DNA methylation inhibits transcription elongation in *Neurospora crassa* and *Candida albicans* (Rountree et al., 2000; Mishra et al., 2011). In *Magnaporthe oryzae*, DNA methylation is also critical for fungal development and genome defense throughout the asexual life cycle (Jeon et al., 2015). Epigenetics is considered to be correlated with genotype, phenotype, and environment in most eukaryotes. It has also been referred to as an important control mechanism used by fungi to modulate the transcription of genes involved in producing secondary metabolites (Liu et al., 2012).

Since 2014, the quality of the accuracy, read length, and throughput when using third-generation sequencing technology has improved (Wang et al., 2021). Therefore, Oxford Nanopore Technologies (ONT) have been widely used for genomic sequencing of different organisms, such as fungi, to assemble high-quality genomes through ultralong reads (882 kilobase pairs or longer) (Fournier et al., 2017; Senol Cali et al., 2019). In addition, based on the theory of ONT sequencing, methylated cytosines can be directly detected by basecalling using raw sequencing data. This platform might facilitate the decoding of fungal genome-wide DNA methylation; however, there are still few data regarding the detection of EPF genome-wide DNA methylation by ONT.

In this study, we collected EPF isolates in Taiwan. After screening the virulence on *Spodoptera litura*, one *B. bassiana* isolate (Bb-NCHU-157) was selected and subjected to whole-genome sequencing by ONT and next-generation sequencing (NGS). Based on the sequenced Bb-NCHU-157 genome, two putative DNMTases (e.g., Bb-Dim2 and Bb-DCM) were found by a BLASTP search, and their expression patterns were also investigated. To address the aim of this study, the genome-wide DNA methylation levels between different developmental stages, including the conidial and mycelial stages, were compared, and the differentially DNA methylated regions (DMRs) and genes in the DMRs between conidia and mycelia were further investigated. Finally, the ONT-predicted genome-wide DNA methylome of Bb-NCHU-157 was validated by bisulfite (BS) conversion PCR in this study.

## Materials and methods

### Entomopathogenic fungi and genomic DNA extraction

The entomopathogenic fungal isolates used in the test were provided by the Insect Pathology and Pathogen Genomics Lab (National Chung Hsing University, Taiwan). EPFs were recovered from −80°C and cultured on 55 mm (Alpha Plus Scientific Corp.) ¼ Sabouraud dextrose agar (SDA) (HiMedia Co.) plates. The cultured plates were incubated at 25°C for 3 days before harvesting mycelia and 10 days before harvesting conidia with a photoperiod of 24 h of darkness. Mycelia were collected from 10 plates by using an inoculation loop, and 6 plates of conidia spores were harvested by sterilized 0.03% Tween 80 solution (PanReac AppliChem Co.). Mycelia and conidia were checked under a light microscope (Nikon Eclipse Ci-L) and subjected to genomic DNA (gDNA) extractions. Fungal genomic DNA was extracted using a Yeast Genomic DNA Kit (Geneaid, TW) and then further purified as following step. Fungal gDNA was mixed with an equal amount of Phenol:Chloroform:Isoamyl Alcohol (25:24:1, MERCK, United States) and centrifuged at 4°C, ×12,000 *g* for 15 min. The supernatant was transferred to a new tube, and the phenol: chloroform: isoamyl alcohol wash was repeated. The supernatant was mixed with 0.1 volume of 3M NaOAC (pH 5.2), and the same amount of isopropanol (Sigma-Aldrich, USA) at −20°C overnight. After centrifuging at 4°C, ×12,000 *g* for 30 min, gDNA was washed with 75% ethanol (Sigma-Aldrich, United States) and centrifuged at 4°C, 12,000 g for 10 min. Once removing the remaining ethanol, the pellet was dissolved in TE buffer. The fungal genomic DNA samples were subjected to next-generation sequencing and nanopore sequencing.

### NGS and data preprocessing

Fungal gDNA was subjected to sequencing library construction. The sequencing library was prepared using a standard protocol from the NEBNext Ultra II DNA Library Prep Kit for Illumina (NEB, USA) and sequenced by an Illumina HiSeq sequencer with a paired-end (PE) technology of 2 × 150 bp (Macrogen, Korea). Total PE reads were subjected to sequencing adapter identification and were then trimmed by cutadapt (Martin, 2011). Ambiguous bases and bases with lower quality values were removed by PRINseq (Schmieder and Edwards, 2011) from either the 5′- or 3′-ends. The final high-quality reads were selected using the NGS QC Toolkit (Patel and Jain, 2012) with default parameters. These trimmed reads were then subjected to genome assembly and annotation by bioinformatics analysis.

### ONT library preparation and sequencing

A total sample quantity of 2 μg of high molecular weight genomic DNA was used for ONT library construction. The ONT sequencing library was prepared from the genomic DNA following the standard protocol with a Ligation Sequencing Kit SQK-LSK109 (Oxford Nanopore Technologies, Cambridge, UK). For reference genome assembly, two libraries (for mycelium and conidia) were sequenced in R9.4.1 SpotON flow cells, then sequenced on a MinION device using ONT MinKNOW software version 22.05.5. At least three gigabases (Gb) of data were obtained from each sequencing run to obtain approximately 80–100 coverages for genome assembly. There are total three replications of ONT sequencing were performed and subjected to the analysis of DNA methylome and differentially DNA methylated regions (DMRs).

### Genome assembly, annotation, and repeat region prediction

The genomic assembly pipeline is summarized in Supplementary Figure S1 and the all code used for the analysis were uploaded in GitHub (https://github.com/IPGyihsuan/Bb-NCHU-157). Quality trimming and adapter clipping of the NGS reads were performed by using Trimmomatic (v0.39) (Bolger et al., 2014). FAST5 files with ONT sequencing reads were basecalled by Guppy (v 6.1.7), obtained from the ONT community site (https://community.nanoporetech.com) (Technologies, 2020), for further analysis, and the barcodes and adapter reads were trimmed from the basecalled reads with Porechop (Wick et al., 2017) (v0.2.4, https://github.com/rrwick/Porechop). Genome assembly was combined with the NGS and ONT sequencing reads by using MaSuRCA (v4.0.6) (using default parameters) (Zimin et al., 2017). To assess the quality and information of the genome assembly, the assembled genome was summarized by using QUAST (v5.1.0) (Gurevich et al., 2013) (Supplementary Figure S1). Fungal genome annotation was performed by using FunGAP (Min et al., 2018). Based on the NCBI relatives’ proteome database and predefined species model of the Augustus species model, the RNA-seq data (SRR3105334, Accession number: PRJNA309019) from the NCBI were used for genome annotation (Supplementary Figure S1). The secondary metabolites were annotated by antiSMASH (Blin et al., 2019). To identify the transposable element (TE) regions, repeatModeler2 (Flynn et al., 2020) and RepeatMasker (Chen, 2004) were used.

### Expressions of DNA methyltransferase in Bb-NCHU-157

Mycelium and conidia were cultured as mentioned above, and the total RNA of the mycelium and conidia stages were extracted by a Presto™ Mini RNA Yeast Advanced Kit (Geneaid, TW). A total of 1 μg of total RNA was used as a template for reverse transcription (RT) using an iScript™ cDNA Synthesis Kit (BIO-RAD, USA) following the manufacturer’s instructions. The synthesized cDNA was used for quantitative PCR to detect DNMTs in the mycelium and conidial stages. To search for DNMTs in the *B. bassiana* NCHU-157 genome, the conserved protein domains of DNMTs were aligned using the Conserved Domain Database (CDD) with default parameters (Marchler-Bauer et al., 2015; Lu et al., 2020) and served as a probe to perform tBLASTn. An e-value lower than 10^−4^ was set as the criterion for DNMT homology (Supplementary Figure S1). Two putative DNMTs (e.g., *Dim-2* and *C-5 cytosine-specific DNA methylase*) were found in Bb-NCHU-157 and selected for further analysis by qRT‒PCR. The specific primers used for qRT‒PCR are listed in Supplementary Table S1. The qRT PCR contained 1 μL of cDNA, 1 μL of forward primer (10 μM), 1 μL of reverse primer (10 μM), 10 μL of iQ SYBR Green Supermix (BIO-RAD, USA), and 7 μL of ddH_2_O. The qRT‒PCR program was conducted as follows: 1) 95°C for 90 s, 2) 95°C for 15s, 3) 60°C for 30 s, 4) steps 2) to 3) were repeated for 40 cycles, and 5) 72°C for 30 s. *Bb γ-actin*, which has been shown to be stably expressed under a variety of conditions (Yang et al., 2016), served as an internal control to normalize the relative expression levels. The relative gene expression levels were calculated by using the 2^−△Ct^ method (Livak and Schmittgen, 2001). A total of three replicates were performed independently for each gene in each life stage.

### Analysis of the DNA methylome and DMRs

A flowchart of the DNA methylation analysis is summarized in Supplementary Figure S2 and the all code used for the analysis were uploaded in GitHub (https://github.com/IPGyihsuan/Bb-NCHU-157). DNA methylation analysis was performed using DeepSignal-plant (Liu et al., 2021). FAST5 files of conidia and mycelium were subjected to DeepSignal-plant analysis based on the genome assembled in this study as the reference genome. The multiple reads contained in each FAST5 file were first converted to a single-read FAST5 file by multi_to_single_fast5 provided by ONT (https://github.com/nanoporetech/ont_fast5_api), and the single-read FAST5 file was then basecalled using Guppy (v 6.1.7). The tombo preprocess (using default parameters) and tombo reguiggle (using default parameters) were used to preprocess the FAST5 file and basecalled file. After tombo preprocessing, the “model.dp2.CNN.arabnrice2-1_120m_R9.4plus_tem.bn13_sn16.both_bilstm.epoch6.ckpt” model, which was provided by DeepSignal-plant, was used as the deepsignal_plant call_mods command to call the methylation, and the script, split_freq_file_by_5 mC_motif.py, which was provided by DeepSignal-plant, was then used to identify the methylation frequencies of CG, CHH and CHG. The methylation frequency results were visualized by R (v 4.2.0).

To identify the DMRs, a non-overlapping tiling window approach was used. Regions containing at least five cytosines, each of which was covered by at least four reads in both the mycelium and conidia samples, were included in the analysis. To be considered a DMR, a region had to show 2-fold differences in global methylation levels with the criteria of *p-*value < 0.05 in the CG, CHG and CHH contexts. Genes overlapping with DMRs in the promoters (e.g., −1,000 bp to + 500 of TSS) were recognized as differentially methylated genes (DMGs) and the DMGs were further subjected to gene ontology (GO) enrichment analysis by using Blast2GO (Gotz et al., 2008).

### Bisulfite conversion and PCR validation

A total of five DMRs were randomly selected for bisulfite PCR (BS-PCR) validation. The mycelium and conidia stages were cultured as described above. Total genomic DNA samples from these cultures were subjected to BS-PCR by using an EpiMark Bisulfite Conversion Kit (NEB) following the instructions in the user manual. Briefly, DNA (1 μg) was BS converted, and the BS-converted DNA samples were subjected to desulfonation and cleanup with an EpiMark spin column (NEB). A total of 3 μL of BS-converted DNA was PCR-amplified by EpiMark Hot Start Taq DNA Polymerase (NEB) with the following PCR program: 1) 95°C for 30 s, 2) 95°C for 15 s, 3) 50°C for 30 s, 4) 68°C for 1 min, 5) steps 2) to 4) were repeated for 40 cycles, and 6) 68°C for 5 min. The primers used for BS-PCR are listed in Supplementary Table S1. The amplicons were analyzed by electrophoresis in a 1.5% agarose gel, and the bending signals were semi-quantified by ImageJ Version 1.53 t. The purified amplicons were then sequenced directly (Genomics Co., TW).

## Results

### Sequencing data summary

The NGS library summary and ONT sequencing raw data for reference genome assembly are listed in Supplementary Tables S2, S3, respectively. The raw data obtained from next-generation sequencing contained 2.9 million M) reads with 4.7 Gb (Supplementary Table S2)**.** For NGS, a total of 2.2 M quality reads (74.7%) with 3.8 Gb (>Q30) (80.9%) were trimmed by using Trimmomatic (Bolger et al., 2014) and subjected to final genome assembly (Supplementary Table S2). The ONT data used for genome assembly were basecalled using Guppy and then quality checked and trimmed by using Porechop (v0.2.4) (R. Wick. 2017). Two different fungal life stages were subjected to ONT, which generated 2.8 M reads with 2.9 Gb and 2.6 M reads with 3 Gb for conidia and mycelium, respectively. After trimming, 2.7 M reads (96.7%) with 2.8 Gb (>Q30) (97.2%) and 2.3 M reads (89.5%) with 2.9 Gb (>Q30) (99.1%), respectively, were integrated for genome assembly and DNA methylome analysis (Supplementary Table S3). In summary, a total of 2.2 M reads from 3.8 Gb from the NGS data and 6.3 M reads (97.4%) with 5.8 Gb (98.9%) from the ONT data were subjected to the assembly approach.

### Genome assembled of Bb-NCHU-157

**FIGURE 1:**
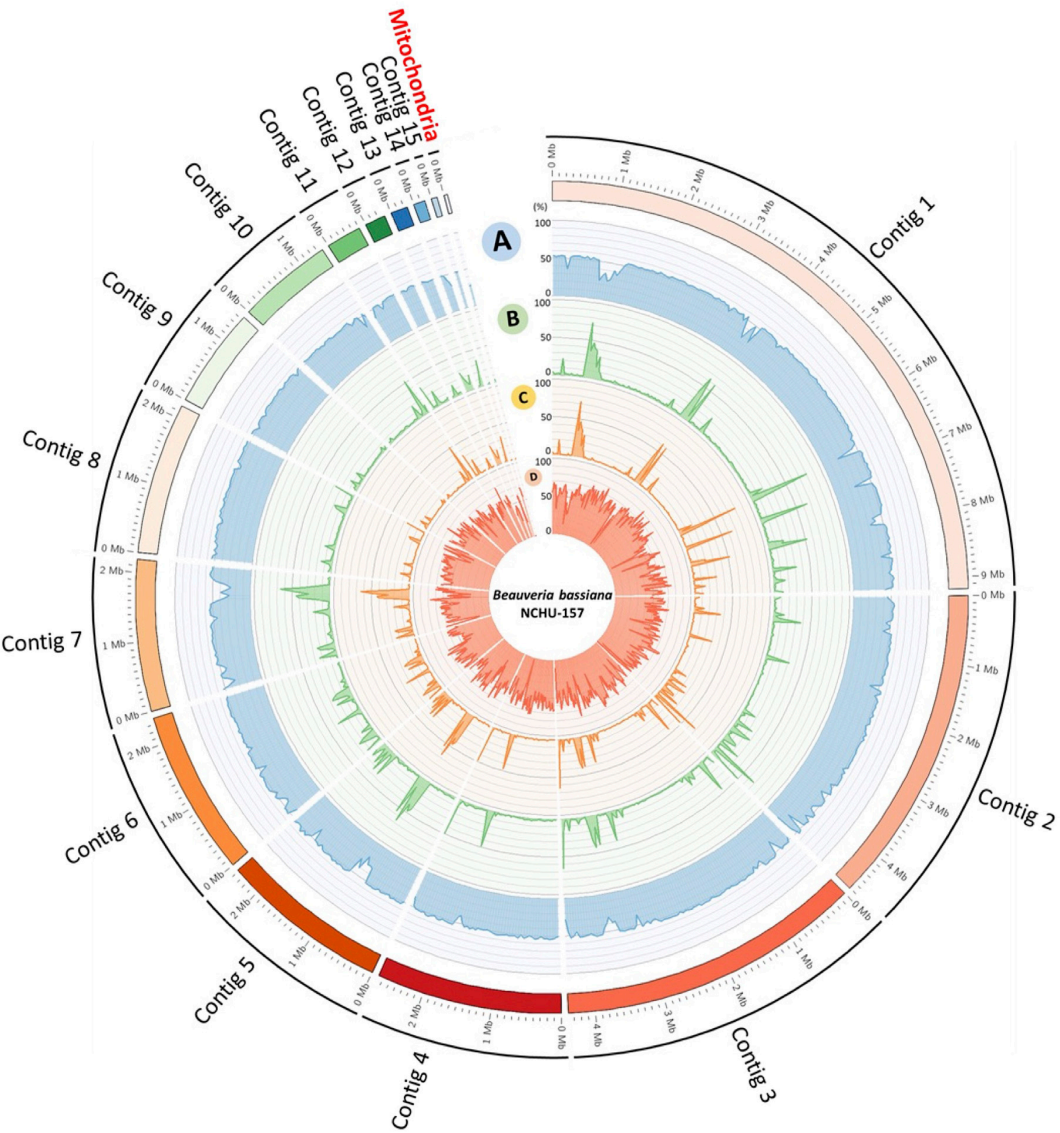
Whole genome map of fifteen core chromosomes plus one mitochondrial genome of Bb-NCHU-157. **(A)** GC%, **(B)** repeat density, **(C)** TE density, and **(D)** gene density. All skew CIRCOS maps were drawn with a 50 kbp window.

The information of the genomes assembled from two different fungal developmental stages, including conidia and mycelium, based on the ONT data were summarized in Supplementary Tables S4, S5. To obtain a better reference genome for DNA methylome analysis and improve the genome assembly performance, the NGS data were integrated into the final genome assembly of Bb-NCHU-157. The final genome of Bb-NCHU-157 was presented as Circos plots, which show the percentages of GC content, repeat regions, TEs and gene proportions in each contig in every 50 kbp window (Figure 1). The assembled whole genome consisted of sixteen contigs with a GC content of 50.43%. The final genome of Bb-NCHU-157 had a total length of 34.19 Mbp (Table 1; Figure 1). Among these contigs, the largest contig was 9.18 Mbp, and the N50 contig size was 4.38 Mbp (Table 1). FUNGAP annotation of the assembled genome identified 10,848 protein-coding genes with a gene density of 54.12% (317 genes/Mb) (Table 1; Figure 1). The number of exons was 28,214, with an average of 2 exons per gene (Table 1). In the Bb-NCHU-157 genome, repeat region positions were predicted as 6.48%, and the TEs were predicted as 4.48% (Table 1; Figure 1). It was noted that the regions with lower GC contents had higher proportions of repeat sequences, TE distributions and lower gene densities, indicating that these gene regions in the genome might be more stable or unchangeable than the repeat regions (Figure 1).

**TABLE 1:** Genome assembly of *Beauveria bassiana* (Bb-NCHU-157).

Assembled genome	Combined genome
contigs	16
Largest contig (bp)	91,76,171
Total length (bp)	3,41,89,895
Coverage	75-84X
GC (%)	50.55
N50 (bp)	43,80,399
L50	3
N90	14,43,135
L90	9
Total protein coding genes	10,848
Gene density (gene/Mb)	317.29
Number of exons	28,240
Number of exons per gene (med)	2
mitochondria genome size (bp)	56,240
Number of tRNA	161
Number of secondary metabolize	41
TE density	4.48

### Expressions of DNMTs in different life stages of Bb-NCHU-157

**FIGURE 2:**
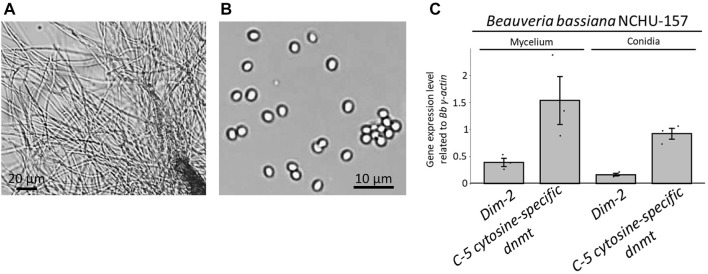
Two life stages of Bb-NCHU-157 and the relative gene expression levels of two DNMTs (e.g., *Dim-2* and *C-5 cytosine specific DNA methylase*) in two different life stages of Bb-NCHU-157. **(A)** Mycelium stage with 3 days of culture on ¼ SDA plates, **(B)** conidia stage with 7 days of culture on ¼ SDA plates, and **(C)** relative gene expression levels of *Dim-2* and *C-5 cytosine-specific DNA methylases* in the mycelium and conidial stages.

The morphologies of the two different life stages of Bb-NCHU-157 were observed (Figures 2A, B). The gene expression levels of the two DNMT genes (e.g., *Dim-2* and *C-5 cytosine-specific DNA methylase*) were explored in two different life stages, including mycelium and conidia (Figures 2A, B). Both DNMTs showed higher expression levels in the mycelium stage than in the conidial stage (Figure 2C), indicating that DNA methylation development in *B. bassiana* might occur in the mycelium stage. Among the two DNMTs, the *C-5 cytosine-specific DNA methylase* showed much higher expression levels than *Dim-2* in both life stages, suggesting that the *C-5 cytosine-specific DNA methylase* might play an important role in DNA methylation in *B. bassiana.*

### DNA methylomes of Bb-NCHU-157

**FIGURE 3:**
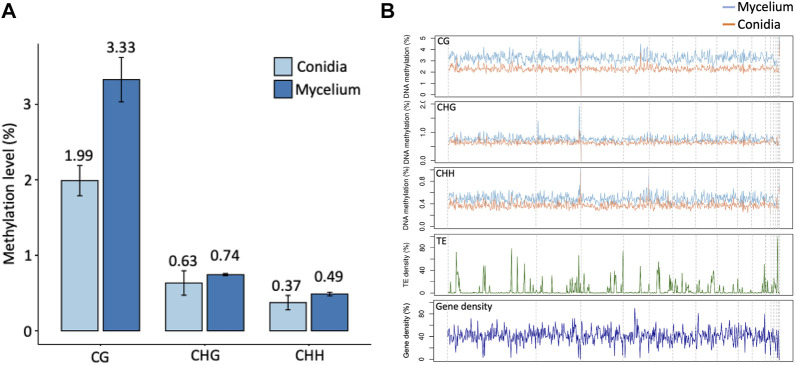
Global patterns of DNA methylation in Bb-NCHU-157. **(A)** Average methylation levels in conidia and mycelium. **(B)** CG, CHG, and CHH methylation levels in Bb-NCHU-157 genome and the densities of Transposable elements (TE) and genes for the same contigs.

**FIGURE 4:**
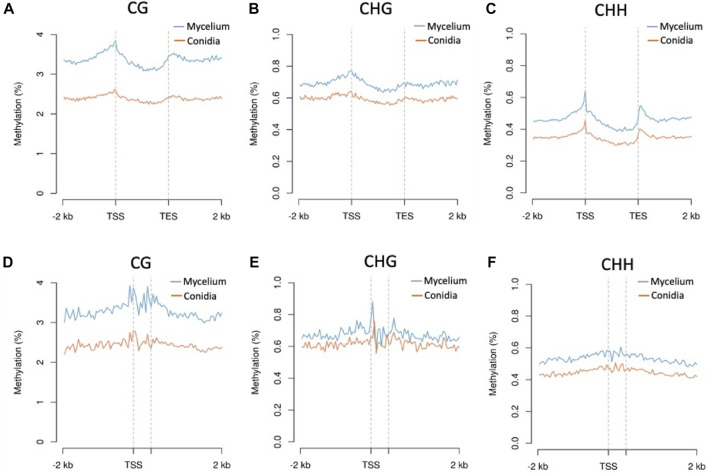
Methylation levels of the CG, CHG, and CHH contexts of conidia and mycelium in the Bb-NCHU-157 genome. Densities of the CG, CHG, and CHH contexts around genes and transposons are shown using MetaPlot. Conidial and mycelial methylation levels around genes **(A**–**C)** and transposable elements **(D**–**F)**. TSS, transcription start site and TES, transcription end site.

Three replications of ONT sequencing data were used for the DNA methylome analysis (Supplementary Table S6). The global DNA methylation levels of the mycelium stage (5 mC = 3.44%) were higher than those of the conidial stage (5 mC = 2.06%) (Figure 3A). Three different methylation contexts were also identified, including CG, CHG and CHH, by the DeepSignal-plant model (Liu et al., 2021). In summary, the Bb-NCHU-157 genome revealed CG methylation preferences, which were 3.33% and 1.99% in the mycelium and conidial stages, respectively, while lower methylation frequencies were found in the CHG (mycelium = 0.74% and conidia = 0.63%) and CHH contexts (mycelium = 0.49% and conidia = 0.37%) (Figure 3A). Analysis of the methylation locations indicated that the DNA methylation pattern revealed highly coordinated localization with the TE regions but was not located in the gene regions at all three methylation contexts, indicating a general pattern of the fungal methylome (Figure 3B). Further investigation of the DNA methylation tendencies in the gene regions and TE regions in the Bb-NCHU-157 genome showed that a preference for DNA methylation was found in the TE regions rather than in the gene regions (Figure 3; Supplementary Figure S3). In the gene regions, the methylated locations were mainly observed in the TSS and TES in all three CG, GHG and CHH contexts, while the TE regions showed high methylation levels (Figure 4). Although low methylation levels were found in the CHG site of both the mycelium and conidial stages, they showed a more obvious pattern of TSS and TES methylation in the gene regions and TE methylation (Figure 4).

### Identification of DMRs

**FIGURE 5:**
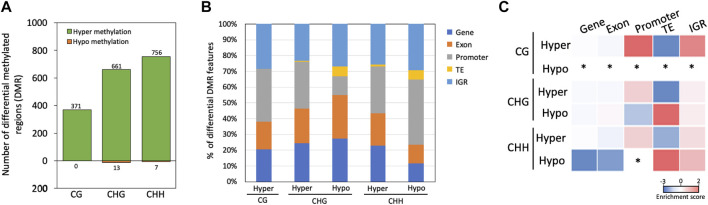
Identification and characterization of differentially methylated regions between conidia and mycelium in Bb-NCHU-157. **(A)** Hypermethylated and hypomethylated regions based on a comparison of the mycelium and conidial stages; **(B)** proportions of different methylated contexts that are hypermethylated and hypomethylated in different regions; and **(C)** enrichment analysis of DMRs. * = nd.

Most of the DMRs were hypermethylated in the mycelium stage, including 371, 661 and 756 DMRs in the CG, CHG, and CHH contexts, respectively; only 13 and 7 DMRs were hypomethylated in the mycelium in the CHG, and CHH contexts, respectively, while no any hypomethylated region was detected in the mycelium in the CG context (Figure 5A). Moreover, CG and CHG methylation mainly occurred in the promoter and intergenic region (IGR) with hypermethylation in the mycelium, while CHG and CHH methylation was located in the TE region in hypomethylated mycelium (Figures 5B, C). In the methylation enrichment analysis, both CHG and CHH showed a much higher enrichment score in hypomethylation (CHG enrichment score = 1.34; CHH enrichment score = 1.67) than hypermethylation of mycelium (CHG enrichment score = −2.37; CHH enrichment score = −0.68) (Figure 5C).

### GO analysis

**FIGURE 6:**
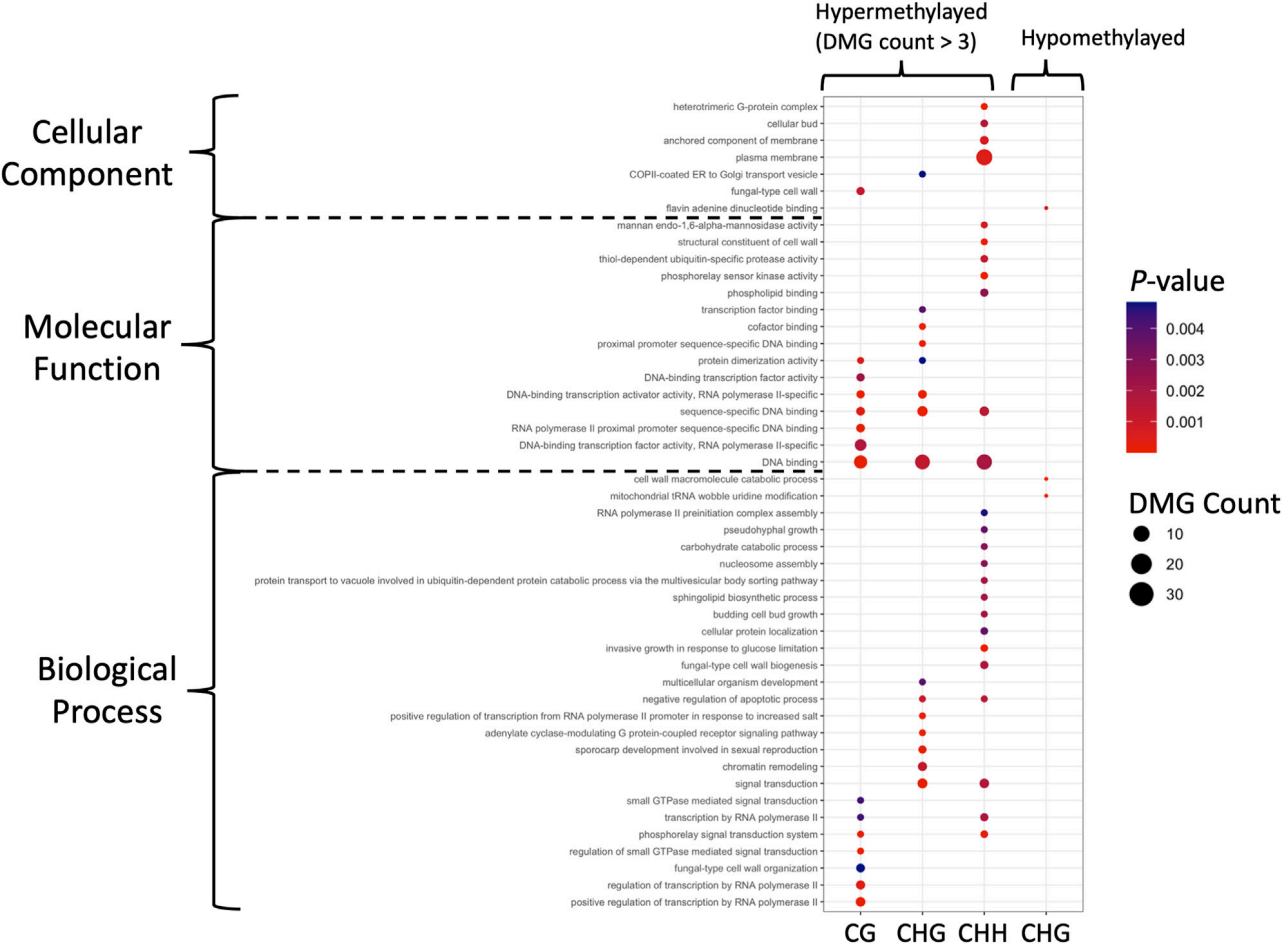
GO identification of the DMGs in DMRs. Hypermethylated and hypomethylated DMGs with *p*-value < 0.05 are presented as dot plots. Each methylation pattern is indicated at the bottom. Colors indicate the *p-*values obtained from Fisher’s exact test, and the dot sizes are proportional to the number of DMGs involved in the GO pathway.

Similar to the DMRs, the DMRs were mostly hypermethylated in the mycelium stage, including 241, 414 and 446 DMGs in the CG, CHG, and CHH contexts, respectively; only 3 DMGs were hypomethylated in the mycelium in the CHG context. Genes located in the DMRs were subjected to GO analysis. The DMGs were classified into three GO categories, including biological process (BP), cellular components (CC) and molecular functions (MF) (Supplementary Tables S7–S10). Compared to the DMGs that were hypermethylated in the mycelium, DMG hypomethylation revealed a different pattern (Figure 6). For conidia hypomethylation, only the CHG site was identified in the GO categories of CC and BP, respectively (Figure 6). The GO term classification results showed that the DMGs shared common GO terms of DNA binding (GO:0003677), and sequence-specific DNA binding (GO: 0043565) in the CG, CHG and CHH contexts for mycelium hypermethylation (Figure 6; Supplementary Tables S7, S8, S10). In addition, the hypermethylated DMGs were highly involved in protein dimerization (GO: 0046983) and DNA-binding transcription factor activity, RNA polymerase II-specific (GO: 0000981) for CG/CHG in the MF and negative regulation of apoptotic process (GO: 0043066) and signal transduction (GO: 0051056) for CHH/CHG in BP, and transcription by RNA polymerase II (GO: 0006357) and phosphorelay signal transduction system (GO: 0000160) for CG/CHH in BP (Figure 6). In contrast, only three hypomethylation of GO terms were identified in CHG, including Flavin adenine dinucleotide binding (GO: 0050660) in CC, cell wall macromolecule catabolic process (GO: 0016998) and mitochondrial tRNA wobble uridine modification (GO: 0070899) in BP (Figure 6; Supplementary Table S9).

### Validation of DNA methylation

**FIGURE 7:**
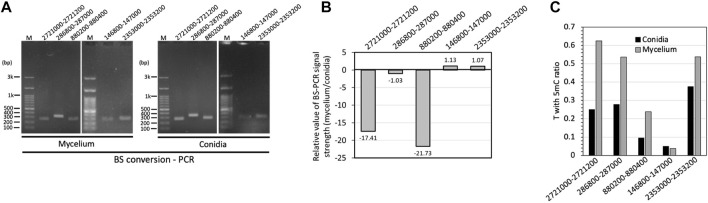
Validation of DMRs among two fungal developmental stages by BS conversion PCR. **(A)** DNA electrophoresis gel of PCR amplified from BS converted mycelium and conidia DNA samples. **(B)** Semiquantitative BS-PCR based on the relative values of mycelium to conidia. **(C)** Ratios of T with C signal in Chromatograms of Sanger sequencing in mycelium and conidia.

To further confirm the results of the methylome predictions obtained from the Nanopore data, we performed BS-PCR and sequenced five selected DMRs for validation. Based on the results, three BS-converted DNA fragments obtained from conidia showed stronger bending signals than those from mycelium, while two DMRs showed hypomethylated patterns in mycelium, indicating a higher methylation level in the mycelium genome, which supported the same correlation with the nanopore-based DNA methylome prediction (Figures 7A, B). Although the Sanger sequencing results showed the sequences of cytosine (C) to thymine (T), the chromatograms revealed mixed T and C signals (Figure 7C; Supplementary Figure S4), suggesting that unconverted 5 mC bases exist in the DMRs.

## Discussion

Combining the sequence reads from ONT and Illumina for genome assembly with MaSuRCA could obtain a highly contiguous, accurate genome (Zimin et al., 2013; Zimin et al., 2017). In this study, both ONT datasets (conidia and mycelium) showed high quality for the longest reads (longer than 1 Mb) (Supplementary Table S4). Furthermore, the hybrid genome exhibited high quality in terms of the number of contigs, largest contigs and N50 (4.38 Mbp), indicating that the combination of long reads (ONT data) and short reads (NGS data) showed better genome assembly performance.

The longest contig length in the hybrid genome was 9.18 Mbp, which was longer than that of the ONT sequence-only genome based on our data. In addition, the number of putative protein-coding genes is similar to that of the *B. bassiana* reference genome in the NCBI (RefSeq GCF_000280675.1). The *B. bassiana* genome was assembled based on the sequences obtained from the Illumina data and PacBio RS II platform (Xiao et al., 2012; Lee et al., 2018). The genome assembled with Illumina data showed a shorter N50 (0.73 Mbp) than that of PacBio (3.12 Mbp), and both the Illumina data and PacBio RS II data assembly genomes were shorter than that of the hybrid genome (N50 = 4.38 Mbp) in this study, suggesting the high quality of this genome assembly strategy, and the hybrid Bb-NCHU-157 genome is presumed to be a better reference genome for further annotation and DNA methylome analyses.

DNMTs are a group of enzymes that contribute to DNA methylation by transferring the activated methyl group (CH_3_) from S-adenosyl methionine (SAM) to the 5th position of a cytosine residue (5 mC); therefore, DNA methylation in the genome is correlated with the activities of DNMTs (Law and Jacobsen, 2010). It has been proven that DNMTs showed higher expression levels in the mycelium stage in *Metarhizium robertsii,* and higher methylation levels were also found in the mycelium stage (Li et al., 2017). Our data indicate that the two DNMTs (e.g., *Dim-2* and *C-5 cytosine-specific DNA methylase*) showed higher expression levels in the mycelium stage than in the conidia stage in Bb-NCHU-157, suggesting that the development of DNA methylation in *B. bassiana* might occur in the mycelium stage. Based on our data, the *C-5 cytosine-specific DNA methylase* levels in the two life stages of Bb-NCHU-157 were higher than those of *Dim-2,* suggesting that *C-5 cytosine-specific DNA methylase* might play an important role in DNA methylation in *B. bassiana.* Indeed, it is known that *C-5 cytosine-specific DNA methylase* is a key epigenetic marker in eukaryotes (Rosic et al., 2018). *C-5 cytosine-specific DNA methylase* shares a well-conserved amino acid domain and function within both eukaryotes and prokaryotes; therefore, its general role in methylation is supported across different species (Kumar et al., 1994).

Based on their structural and functional similarities, DNMTs can be divided into five classes (Ponger and Li, 2005; de Mendoza et al., 2019; Nai et al., 2020). Two DNMTs (e.g., *Dim-2* and *C-5 cytosine-specific DNA methylases*) were found in Bb-NCHU-157. *Dim-2* is a subfamily of DNMT1 and is considered a key factor in methylation maintenance (Ponger and Li, 2005; Huang et al., 2016; de Mendoza et al., 2019; Nai et al., 2020). The C-5 cytosine-specific DNA methylase is an integral part of the DNA restriction modification machinery and has unique properties (Kumar et al., 1994; Zingg et al., 1996; Silvestre et al., 2021). In summary, the two DNMTs may cowork on whole genomic DNA methylation in Bb-NCHU-157. The different expression levels of these two DNMTs might result in differences in DNA methylation levels between the two life stages of Bb-NCHU-157.

The methylated DNA bases in the ONT data could be directly detected from the native sequenced reads. DeepSignal is a tool used for analyzing DNA methylation with ONT data. It has been shown that ONT sequence analysis by DeepSignal-plant was correct for BS sequencing in a plant model (Liu et al., 2021). For the DNA methylation analysis, the Tombo (version 1.5.1) (Stoiber et al., 2017) step in DeepSignal-plent consists of mapping the raw ONT electronic signal to the reference genome; therefore, the ONT data are expected to completely map to the reference genome (Liu et al., 2021). Based on our data, high mappabilities of the genomic sequences of two fungal developmental stages to the hybrid reference genome were found, indicating that the performance of this analytical approach could be used further.

To date, approximately fourteen fungi have been described in studies of DNA methylation in different life stages (Nai et al., 2020). In these fungi, lower methylation levels were usually observed in mycelium, which ranged from 0.22% to 1.67% (Selker et al., 2003; Jeon et al., 2015; Wang et al., 2015; Li et al., 2017; Binz et al., 2018). In our study, the methylation levels in mycelium (5 mC = 3.44%) were higher than those in conidia (5 mC = 2.06%) and were even higher than previously reported entomopathogenic fungal methylation levels. Fungal DNA methylation is known to be associated with reproductive structures, dimorphic transitions and phenotypes, and most fungal DNA methylation shows lower methylation levels in the mycelium than in the conidial stage (Nai et al., 2021). In contrast, lower methylation levels in the conidia of Bb-NCHU-157 were found by ONT. Our finding is similar to the genome-wide DNA methylation of *M. robertsii*, which also showed lower methylation levels (0.38%) in conidia than in mycelia (0.42%) by whole genome BS sequencing (Li et al., 2017). In terms of the methylated locations in the Bb-NCHU-157 genome, both the gene and TE regions could be detected with different methylation patterns (CG, CHG and CHH), while TE methylation is preferred in the Bb-NCHU-157 genome rather than in gene regions. Compared to *M. robertsii*, a similar tendency of higher methylation levels in the mycelium stage was also found in the gene regions of Bb-NCHU-157. Instead of a CHH site preference in the TES in *M. robertsii*, Bb-NCHU-157 gene region methylation was found with methylation in the CG, CHG and CHH contexts and strong preferences in the TSSs and TESs of the gene regions, suggesting that the methylated CG islands at TSSs might tend to block the initiation of transcription after DNA has been assembled into nucleosomes (Jones, 2012; Li et al., 2017). In summary, from the abovementioned results, a lower methylation level in conidia might be conserved in the EPF group, and higher methylation in mycelium might play an important role in regulating fungal cell division and growth.

TEs are repetitive DNA sequences that generate intra- and interspecific genetic variability. To protect against the effects caused by TE movement and replication, including chromosomal rearrangements, deletions, duplications, and regulatory changes, fungi use several mechanisms, one of which is methylation (Dallaire et al., 2021). In terms of TE methylation in Bb-NCHU-157, mycelium also revealed higher methylation levels than the conidial stage. In addition, in the DMR enrichment analysis, TE region methylation strongly occurred in the CHH site, indicating that the stabilization mechanism is highly involved in the mycelium stage. In *Pleurotus ostreatus* and *Pleurotus tuoliensis*, the genes located inside TE-rich clusters showed high methylation levels, and this phenomenon was also observed in *Rhizophagus irregularis*, although the authors also described low methylation levels in older, short, and expressed TEs (Dallaire et al., 2021; Nai et al., 2021). However, hypomethylation of TE in the DMR enrichment analysis was found in the conidia stage of Bb-NCHU-157. It has been reported that hypomethylated or unmethylated TEs were found to be transcriptionally active in *Tuber melanosporum*, implying that lower methylation levels may lead to more complicated epigenetic regulation (Nai et al., 2021). The different methylation levels in the two life stages may affect the regulation of TEs with the development of fungi.

Based on our data, most of the DMGs were found to be hypermethylated in mycelium and shared the common GO terms of DNA binding (GO:0003677), and sequence-specific DNA binding (GO: 0043565) in CG, CHG and CHH contexts, suggesting that the expressions of the groups of genes in these GO categories might be influenced by DNA methylation. Research on *M. robertsii* indicated that moderate methylation of the promoter region tended to increase gene expressions (Li et al., 2017). However, the associations of gene expression and DNA methylation in Bb-NCHU-157 need to be further evaluated. However, our results showed the contrasting life stage methylation levels between *B. bassiana* and other studied fungi, which provide a new direction for discussing methylation levels affecting changes in life stages.

## Data Availability

The NGS sequencing data and ONT sequencing data were deposited in NCBI with BioProject ID: PRJNA895935.

## Supplementary material

Supplementary Figure S1 Genome Assembly, annotation, and DNMT search flowchart of Bb-NCHU-157. Quantity checking of the sequencing data from the NGS and ONT sequencing methods is summarized in Supplementary Tables S1, S2. The whole genomic sequence was annotated by FunGAP in silico.

Supplementary Figure S2 Analysis pipeline of DNA methylation and DMRs of two life stages (conidia and mycelium) in Bb-NCHU-157 for 5mC detection at CpG, CHH and CHG contexts from nanopore sequencing. Approach used to test the Tombo, DeepSignal-Plant, and Guppy tools. All tools predict the methylation frequencies at each genome site from FAST5 input files.

Supplementary Figure S3 Methylation levels of **(A**,**D)** CG, **(B**,**E)** CHG, and **(C**,**F)** CHH contexts in conidia and mycelium in the Bb-NCHU-157 genome. The densities of CG, CHG, and CHH contexts around genes and transposons are shown using MetaPlot. TSS= transcription start site and TES= transcription end site.

Supplementary Figure S4 Validation of DMRs among two fungal developmental stages by BS conversion PCR. **(A)** Sanger sequences of five DMRs original genome sequence; b. Putative BS conversion genome sequence; c. BS converted conidia genome sequence; d. BS converted mycelium genome sequence. The red box indicates T with C signal. The chromatograms of underlined regions were shown in **(B)**. The arrow indicated the BS converted C to T with methylated C signal.
