# Supplementary figures and images for: Whole-genome DNA methylome analysis of different developmental stages of the entomopathogenic fungus Beauveria bassiana NCHU-157 by nanopore sequencing
Source: Front Genet. 2023 Jan 18;14:1085631. doi: 10.3389/fgene.2023.1085631 (PMC9889659; doi:10.3389/fgene.2023.1085631)

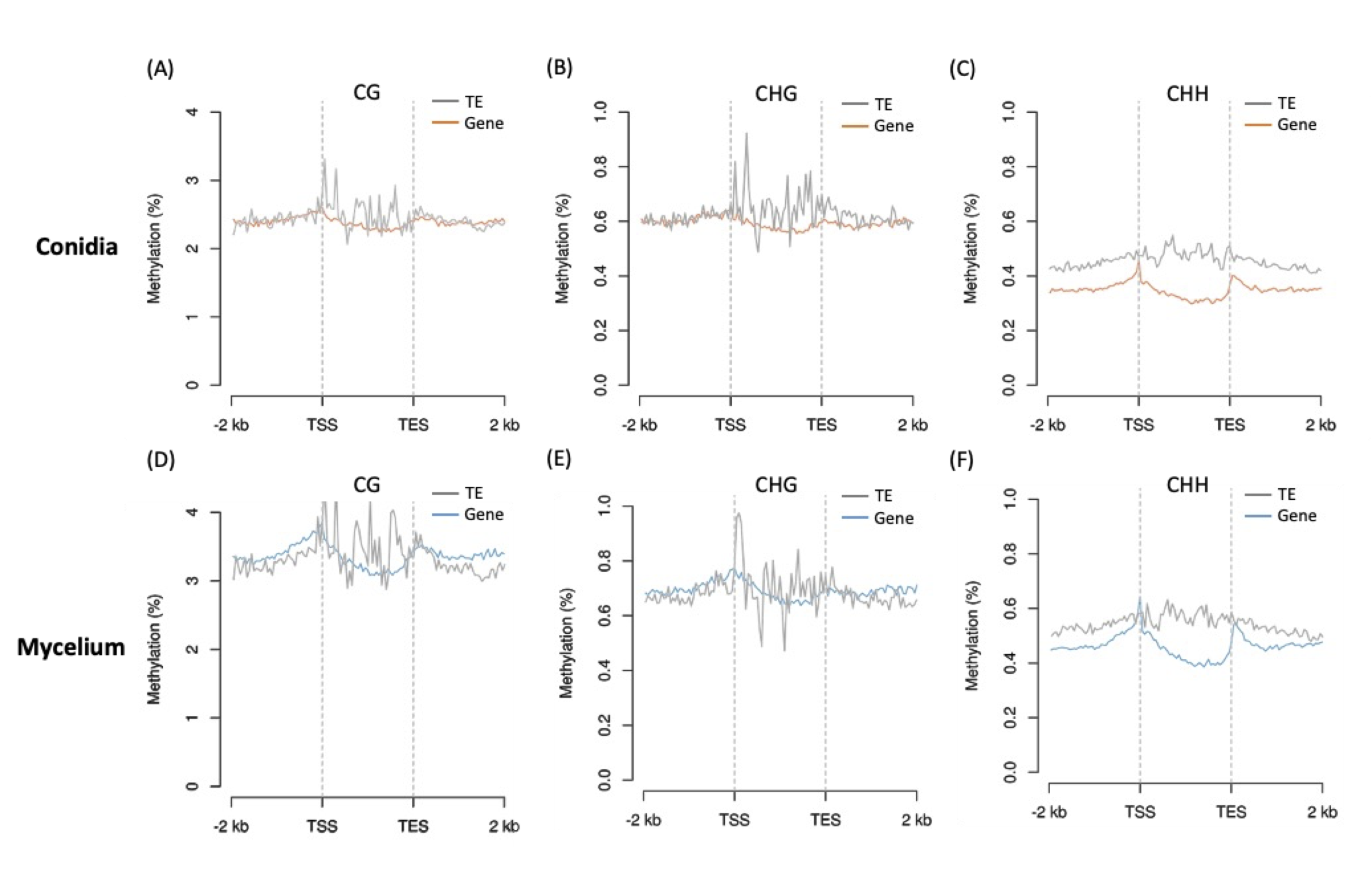

Supplement: Supplementary file 1 [file Image3.TIFF]

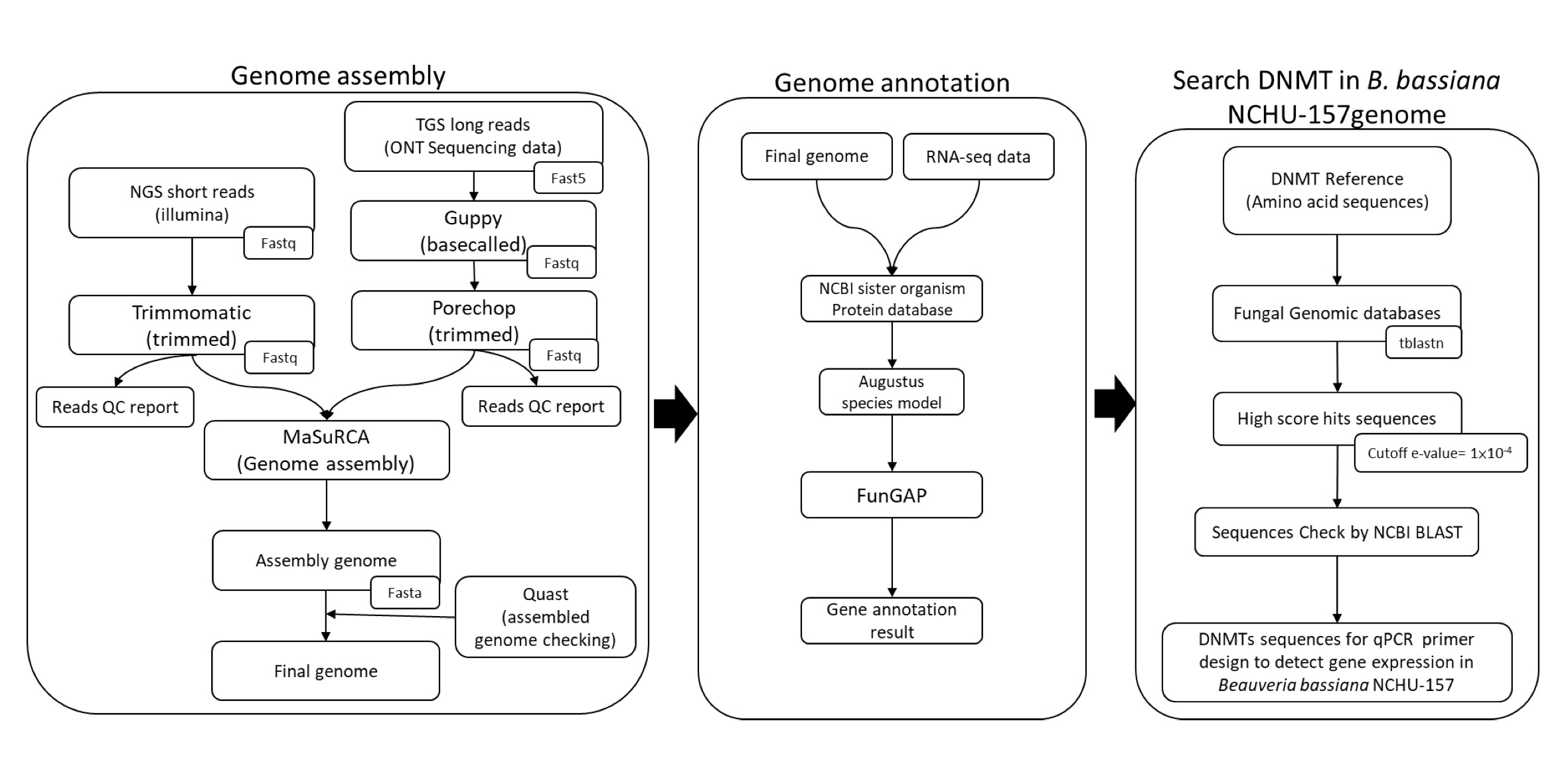

Supplement: Supplementary file 2 [file Image1.JPEG]

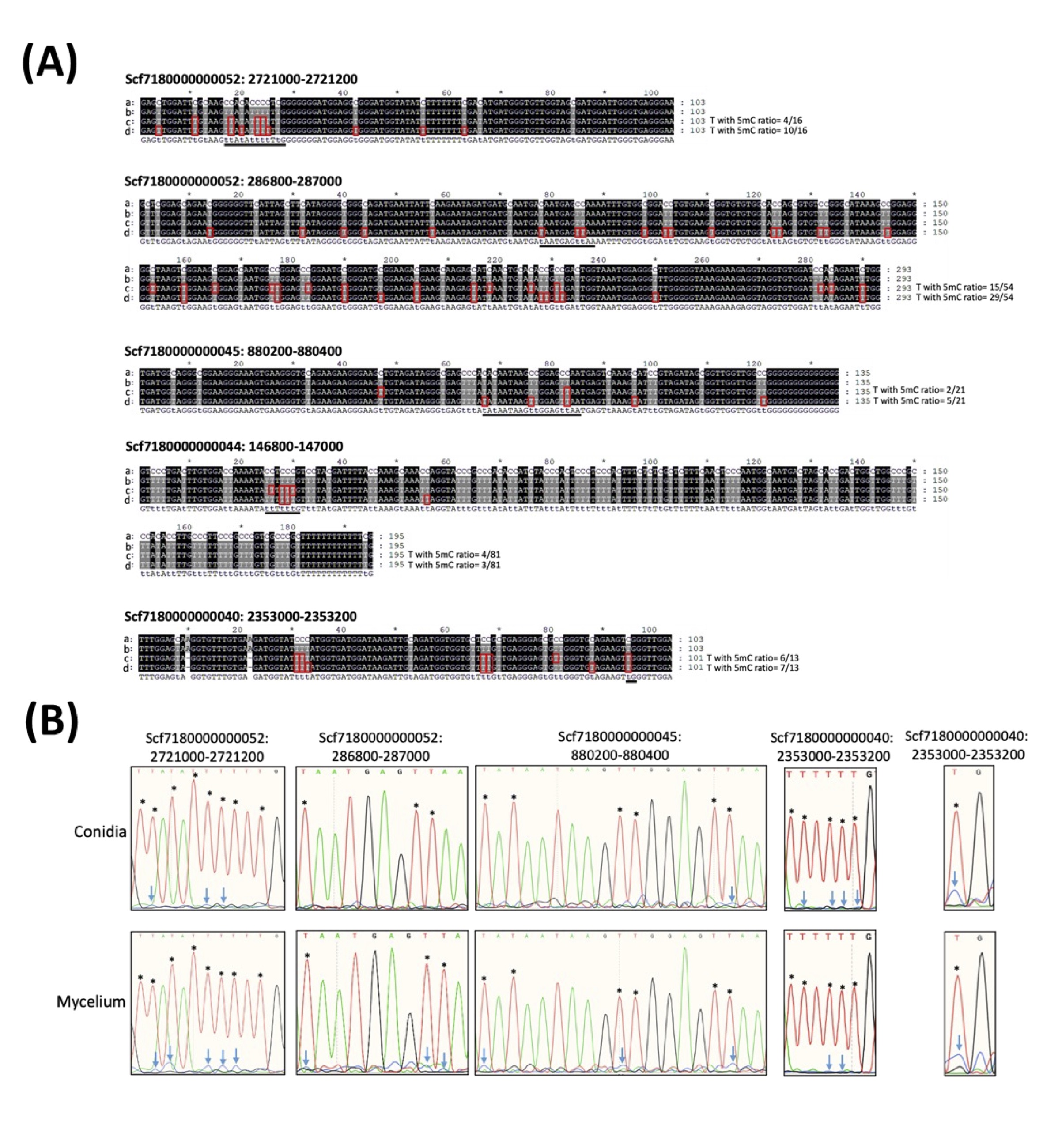

Supplement: Supplementary file 3 [file Image4.JPEG]

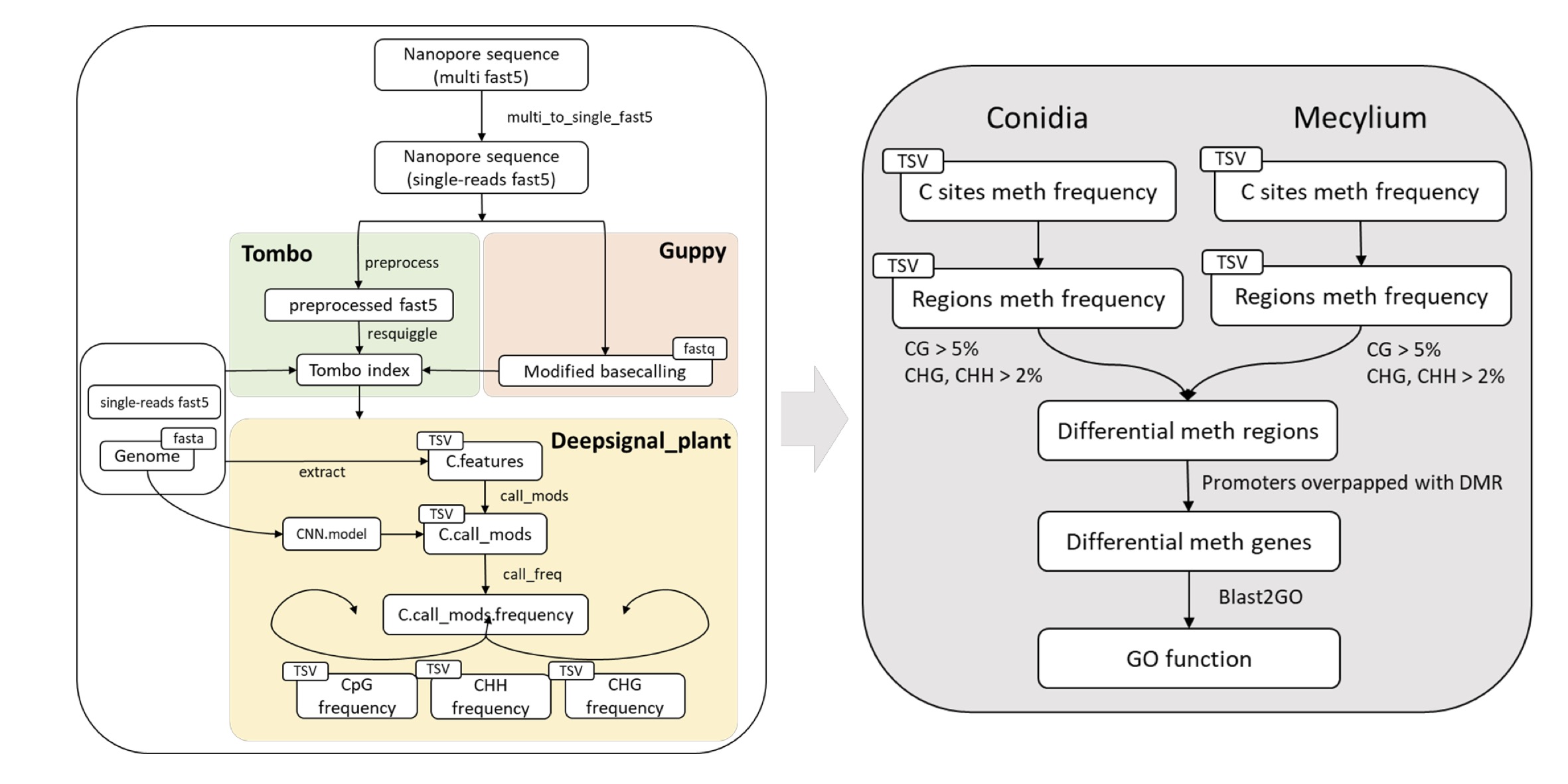

Supplement: Supplementary file 4 [file Image2.JPEG]
